# Safety and efficacy of losartan for the reduction of brain atrophy in clinically diagnosed Alzheimer's disease (the RADAR trial): a double-blind, randomised, placebo-controlled, phase 2 trial

**DOI:** 10.1016/S1474-4422(21)00263-5

**Published:** 2021-11

**Authors:** Patrick Gavin Kehoe, Nicholas Turner, Beth Howden, Lina Jarutyte, Shona Louise Clegg, Ian Brian Malone, Josephine Barnes, Casper Nielsen, Carole Hélène Sudre, Aileen Wilson, Ngoc Jade Thai, Peter Sinclair Blair, Elizabeth Coulthard, Janet Athene Lane, Peter Passmore, Jodi Taylor, Henk-Jan Mutsaerts, David Lee Thomas, Nick Charles Fox, Ian Wilkinson, Yoav Ben-Shlomo, RADAR investigators, Kirsty Harkness, Tarun Kuruvilla, Rupert McShane, Peter Connelly, Gordon Duncan, Lucy Calvert, Alasdair Lawrie, Matthew Sheridan, Eric Jackson, Bernard Udeze, Stephen Pearson, Tobias Langheinrich, Suvarna Wagle, Joseph Butchart, Ajay Macharouthu, Andrew Donaldson, Wendy Neil, Vivek Pattan, David Findlay, Alan Thomas, Robert Barber, Andrew Byrne, Madhusudan Dalvi, Rashi Negi, Bernadette McGuinness

**Affiliations:** a Dementia Research Group, University of Bristol, Bristol, UK; b Dementia Neurology Research Group, University of Bristol, Bristol, UK; c Translational Health Sciences, Population Health Sciences, University of Bristol, Bristol, UK; d Bristol Trials Centre, University of Bristol, Bristol, UK; e Faculty of Health Sciences, Bristol Medical School, Clinical Research Imaging Centre, University of Bristol, Bristol, UK; f Dementia Research Centre, University College London, London, UK; g UK Dementia Research Institute, University College London, London, UK; h UCL Queen Square Institute of Neurology, University College London, London, UK; i MRC Unit for Lifelong Health and Ageing at UCL, and Centre for Medical Image Computing, University College London, London, UK; j School of Biomedical Engineering and Imaging Sciences, Kings College London, UK; k Institute of Clinical Sciences, Queens University Belfast, Royal Victoria Hospital, Belfast, UK; l Amsterdam University Medical Centers, Amsterdam Neuroscience, Amsterdam, Netherlands; m Clinical Pharmacology Unit, School of Clinical Medicine, University of Cambridge, Addenbrookes Hospital, Cambridge, UK

## Abstract

**Background:**

Drugs modifying angiotensin II signalling could reduce Alzheimer's disease pathology, thus decreasing the rate of disease progression. We investigated whether the angiotensin II receptor antagonist losartan, compared with placebo, could reduce brain volume loss, as a measure of disease progression, in clinically diagnosed mild-to-moderate Alzheimer's disease.

**Methods:**

In this double-blind, multicentre, randomised controlled trial, eligible patients aged 55 years or older, previously untreated with angiotensin II drugs and diagnosed (National Institute of Neurological and Communicative Disorders and Stroke and the Alzheimer's Disease and Related Disorders Association criteria) with mild-to-moderate Alzheimer's disease, and who had capacity to consent, were recruited from 23 UK National Health Service hospital trusts. After undergoing a 4-week, open-label phase of active treatment then washout, participants were randomly assigned (1:1) oral over-encapsulated preparations of either 100 mg losartan (after an initial two-dose titration stage) or matched placebo daily for 12 months. Randomisation, minimised by age and baseline medial temporal lobe atrophy score, was undertaken online or via pin-access service by telephone. Participants, their study companions, and study personnel were masked to group assignment. The primary outcome, analysed by the intention-to-treat principle (ie, participants analysed in the group to which they were randomised, without imputation for missing data), was change in whole brain volume between baseline and 12 months, measured using volumetric MRI and determined by boundary shift interval (BSI) analysis. The trial is registered with the International Standard Randomised Controlled Trial Register (ISRCTN93682878) and the European Union Drug Regulating Authorities Clinical Trials Database (EudraCT 2012–003641–15), and is completed.

**Findings:**

Between July 22, 2014, and May 17, 2018, 261 participants entered the open-label phase. 211 were randomly assigned losartan (n=105) or placebo (n=106). Of 197 (93%) participants who completed the study, 171 (81%) had complete primary outcome data. The mean brain volume (BSI) reduction was 19·1 mL (SD 10·3) in the losartan group and 20·0 mL (10·8) in the placebo group. The difference in total volume reduction between groups was –2·29 mL (95% CI –6·46 to 0·89; p=0·14). The number of adverse events was low (22 in the losartan group and 20 in the placebo group) with no differences between treatment groups. There was one treatment-related death per treatment group.

**Interpretation:**

12 months of treatment with losartan was well tolerated but was not effective in reducing the rate of brain atrophy in individuals with clinically diagnosed mild-to-moderate Alzheimer's disease. Further research is needed to assess the potential therapeutic benefit from earlier treatment in patients with milder cognitive impairment or from longer treatment periods.

**Funding:**

Efficacy and Mechanism Evaluation Programme (UK Medical Research Council and National Institute for Health Research).

## Introduction

Alzheimer's disease is currently one of the most costly health-care issues due to a scarcity of effective disease-modifying treatments, which continue to be elusive.^1^ It has been known for several decades that hypertension in mid-life age (50–65 years) increases the risk of developing dementia by 38%,^2^ which might explain how cerebrovascular dysfunction is the earliest detectable pathological event in the development of Alzheimer's disease.^3^ Hypertension and cerebrovascular dysfunction can contribute to reduced cerebral blood flow, loss of cerebrovascular autoregulation, ischaemia, and development of white matter hyperintensities, with the associated loss of cognitive functions that are commonly observed in Alzheimer's disease^4^ as well as Alzheimer's disease-like neurodegeneration.^5^

**Research in context**

**Evidence before this study**

We searched PubMed from database inception to April 30, 2021, for original articles using the keywords “angiotensin” and “Alzheimer” without language restrictions. There is a paucity of randomised controlled trials on hypertension medications that were directly tested in relation to dementia prevention as a primary outcome. A few meta-analyses have analysed data from randomised controlled trials in patients with hypertension or receiving angiotensin-targeting medications for the treatment of stroke, and from several longitudinal population studies that assessed dementia-related factors (eg, some measure of cognitive performance or memory or incidence of dementia) as secondary outcome measures. In several studies, there was evidence supporting a protective effect of angiotensin-targeting medications over other blood-pressure lowering treatments on the development of dementia or of cognitive decline.

**Added value of this study**

To our knowledge, this study is the first to evaluate the potential therapeutic value of an angiotensin II receptor antagonist in Alzheimer's disease using an objective MRI-based primary outcome. This study suggests that 12 months of treatment with losartan is ineffective in reducing the rate of brain atrophy in individuals with mild-to-moderate Alzheimer's disease.

**Implications of all the available evidence**

There is not enough evidence currently to exclude a potential benefit of losartan, or related drugs, if administered for a longer period or in participants with less advanced disease (eg, mild cognitive impairment due to prodromal Alzheimer's disease). Further research is needed to inform whether primary care-based interventions for hypertension and other cardiovascular conditions, which include angiotensin II lowering approaches, might be amenable to primary intervention opportunities to reduce the incidence and progression of Alzheimer's disease, rather than being used as treatments later in the disease course.

Overactivity of angiotensin-converting enzyme (ACE) 1, a component of the classic renin–angiotensin system (RAS), is central to the formation of angiotensin II, a mediator of hypertension, but is also implicated in mechanisms noted in Alzheimer's disease, including neuroinflammation and oxidative stress. Angiotensin II might also contribute to accumulation in human brain tissue of amyloid β (Aβ)-related and tau-related pathologies.^6^ Reduced activity of ACE2, which regulates angiotensin II concentrations in the regulatory RAS pathway, also lends support to the notion of a detrimental involvement of excess concentrations of angiotensin II in Alzheimer's disease.^7^ This hypothesis is supported by preclinical trial findings that showed that enhancement of ACE2 activity in a mouse model of Alzheimer's disease caused a striking reversal of cognitive decline and approximately halved the levels of anticipated Aβ pathology.^7^ Findings during the COVID-19 pandemic indicating how angiotensin II type 1 receptor antagonists (ARAs) might also indirectly promote ACE2 expression, suggest that ARAs might similarly have a benefit in Alzheimer's disease by reducing classic RAS (involved in angiotensin II signalling) activity, while promoting regulatory RAS (involved in angiotensin II metabolism).^8^

Additional mechanisms by which elevated angiotensin II might contribute to Alzheimer's disease are through its reported anticholinergic effects, whereas several preclinical studies have shown that ARAs such as losartan, telmisartan, valsartan, and olmesartan either prevented angiotensin II-mediated Alzheimer's disease-like pathologies in aged rodents or reduced rates of cognitive decline and the extent of pathology in various murine models of Alzheimer's disease.^6^ Collectively, these findings highlight the potential involvement of angiotensin II in several pathological pathways in Alzheimer's disease and frame our core hypothesis that pharmacological intervention against angiotensin II signalling by losartan might provide protection against multiple processes that cause the progression of Alzheimer's disease brain pathology.

Several studies have reported that people taking ARAs have a lower incidence and slower progression of Alzheimer's disease than do those taking other types of anti-hypertensive drugs in individual study populations, although meta-analyses do not support this finding,^9^, ^10^ and yet hypertension management is still viewed as protective.^11^ Losartan, the prototype ARA that inhibited the central actions of angiotensin II in rodents,^12^ has been shown to improve cerebral blood flow, a surrogate marker of cognitive performance in people.^13^ Losartan also limits neuronal damage following ischaemia in rat models of stroke,^14^ and in low doses, given intranasally, reduced neuropathology and improved cognitive performance in transgenic mouse models of Alzheimer's disease, without a blood pressure lowering effect.^15^ Noting the blood pressure lowering function of losartan, it is possible that the drug might have some additional cerebrovascular benefits for patients with Alzheimer's disease, such as reducing the risk of stroke and other forms of cerebral small vessel disease that give rise to white matter hyperintensities. However, we view these as additional benefits rather than the core mechanism from which patients with Alzheimer's disease might benefit from the drug.^16^

Losartan is well tolerated and has advantages over other hypertension treatments in terms of quality of life in older people (age 60–80 years).^17^ Since the involvement of RAS in Alzheimer's disease might be mediated through cardiovascular or non-cardiovascular molecular mechanisms, or both,^17^ losartan might be beneficial in people with Alzheimer's disease who are either hypertensive or normotensive. The Reducing pathology in Alzheimer's Disease through Angiotensin TaRgeting (RADAR) trial was designed to test the hypothesis that blockade of angiotensin II signalling by losartan would slow brain volume loss, as a measure of disease progression, in clinically diagnosed mild-to-moderate probable Alzheimer's disease by reducing brain volume loss as a measure of disease progression.

## Methods

### Study design

This study was a parallel, two-arm, double-blind, placebo-controlled, multicentre, randomised trial done in 23 UK National Health Service hospital trusts. Potential participants were identified through clinic lists or local research registers, or from Join Dementia Research, and some participants self-referred to either their local research team or trial coordination team. A nested qualitative study was also conducted during the trial to explore possible obstacles to recruitment.^18^

The study had two phases. The first phase comprised an open-label active treatment then washout that was undertaken to ensure tolerance of the intervention, particularly for normotensive participants, which was an issue highlighted by patients and carers initially consulted about the intended study. Research has since shown that this issue had an effect on the consideration of hypertension treatments as interventions for dementia.^19^ The second phase was the double-blind randomisation stage.

Potential participants consented to be contacted by their local research team and gave them permission to access their medical records and to speak to their general practitioner to confirm trial eligibility.^20^ Written informed consent was obtained from participants and their study companion. At the time of consent, participants also nominated a legal representative in the event they lost capacity and decisions had to be made at any future point regarding the participant's best interests to remain in the study. Ethics approval for the study and all amendments was given by Wales Research Ethics Committee 2 Cardiff. The UK Medicines and Healthcare products Regulatory Agency also gave authorisation to conduct the trial. There were no major changes to the original protocol (appendix p 1) or the statistical analysis plan during the study. RADAR was overseen by an independent Trial Steering Committee and Data Monitoring and Ethics Committee.

### Participants

Women and men were eligible for this trial if they were clinically diagnosed with mild-to-moderate probable Alzheimer's disease according to the original National Institute of Neurological and Communicative Disorders and Stroke and the Alzheimer's Disease and Related Disorders Association criteria and if they: 1) were aged 55 years or older; 2) had capacity to consent for themselves in accordance with the criteria of the UK 2005 Mental Capacity Act, as judged by trained members of the local research team; 3) had a Mini-Mental State Examination (MMSE) score of 15–28; 4) scored 5 or less on a modified Hachinski scale; 5) had previous CT, single-photon emission computed tomography, or MRI consistent with a diagnosis of Alzheimer's disease; and 6) had a study companion who was willing to participate in the study. Participants could participate regardless of whether they had hypertension and could already be taking licensed anti-dementia treatments and other non-RAS related anti-hypertensive medications.^20^

Patients were deemed ineligible if they met any of the following criteria: 1) potential alternative cause of dementia other than Alzheimer's disease; 2) previous cerebrovascular accident and residual impairment, except for a history of transient ischaemic attack; 3) already receiving ACE inhibitors, ARAs, or potassium-sparing diuretics; 4) known intolerance or renal problems with ACE inhibitors or ARAs; 5) medically unsuitable for, or unwilling, to undergo MRI; 6) consistently low (<115 mm Hg systolic or <70 mm Hg diastolic) or high (>160 mm Hg systolic or >110 mm Hg diastolic) blood pressure in repeated standing and sitting measures intermittently throughout the eligibility visit; 7) a postural drop in blood pressure (a decrease of >20/10 mm Hg on standing from a sitting position, associated with clinically significant symptoms, or a fall of >30/15 mm Hg) at the eligibility visit; 8) impaired kidney function (estimated glomerular filtration rate of <30 mL/min per 1·73 m^2^); 9) evidence of liver disease or significant derangement in liver function test defined as having twice the upper limit of normal for aspartate aminotransferase, alkaline phosphatase, or bilirubin at eligibility visit; 10) other relevant comorbidities, such as hypertrophic cardiomyopathy or clinically significant stenosis of the aortic valve; 11) potassium concentrations higher than 6·0 mmol/L; 12) the potential for pregnancy and unwilling to use effective contraception for the duration of the trial (due to teratogenicity); 13) any reason that would prevent compliance with or completion of the study protocol (eg, terminal illness); or 14) participation in a previous clinical trial of an investigational medicinal product within 6 months of RADAR trial entry.

### Randomisation and masking

Randomisation was contingent on a baseline MRI scan that as part of our quality assurance, was corrected for intensity non-uniformity,^21^ by regularly trained and validated analysts, on scan coverage, per-site protocol consistency, motion, image uniformity, and geometric distortion, based on approaches described elsewhere.^22^ Anonymised scans were uploaded to a bespoke XNAT platform (Cambridge, MA, USA) for secure transfer to Dementia Research Centre University College London (London, UK) and images were assessed according to study identifier, masked to intervention status. If a scan or any requested rescan was of insufficient quality, or participants declined to repeat the scan, they were excluded from randomisation and withdrawn from the study. Participants were randomly assigned (1:1) to either losartan or placebo using an online system or via an automated pin-access telephone service hosted by Sealed Envelope (London, UK; which had no other involvement in the study) using a list generated by the Bristol Trials Centre (Bristol, UK). This randomisation process was implemented by individual recruitment site research staff who received a unique code, which was subsequently passed to local pharmacists who provided the allocated intervention after reconciliation against a protected spreadsheet containing a predefined intervention assignment list. Randomisation was minimised by age (<70, 70–79, >79 years) and visual assessment, by trained and experienced MRI radiographers, of baseline medial temporal lobe atrophy (MTA) score (0–1 [absent or mild], 2–4 [moderate or severe]) according to the Scheltens scale.^23^

This double-blind study meant neither participants, study companions, nor study personnel (except pharmacists at each site) were aware of the treatment allocation. Allocation concealment was achieved using identical over-encapsulated tablets. A 24-h emergency unblinding service was available, if necessary, to all research sites through each local pharmacy service during working hours and out of hours, or through a pharmacy nominated by the RADAR coordination team. Any need for unblinding was adjudicated by the trial manager following consultation with the clinical lead and chief investigator and documented by pharmacy staff and logged centrally. Out of hours or emergency unblinding was possible on request by an attending doctor. If the trial medication was discontinued, participants remained in the study unless they withdrew. Relevant research team members (excluding the trial pharmacist) in any such instances were intended to remain masked, although the success of masking was not formally assessed.

### Procedures

During the open-label phase, participants took 25 mg losartan orally once daily for 7 days and were asked to record their daily blood pressure, using a supplied standard self-administered arm-cuff (Omron, Kyoto, Japan) blood pressure monitor, and any adverse symptoms. Blood samples were collected after 7 days for safety checks (kidney and liver function) and the symptom diary was checked by local researchers in consultation with the investigator where relevant. Participants willing to proceed in the study took 100 mg generic losartan once daily for 7 more days with continued blood pressure and adverse symptom monitoring, after which repeat blood samples were taken. If no contraindications were recorded, participants were given placebo for 4–14 days. This part of the study served as a washout period and tested tolerance to the larger encapsulated tablet and maintained study inculcation for the randomised phase, while allowing time to review blood samples for safety according to exclusion criteria and schedule the baseline MRI.

**Figure 1:**
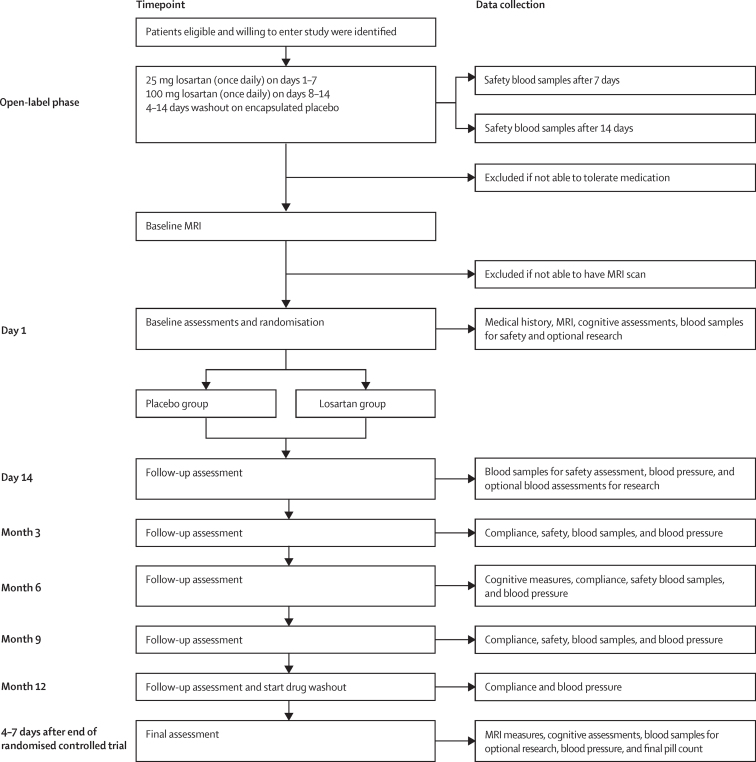
Participant procedures and data collection timepoints Safety blood assessments included measures of electrolytes, creatinine, and liver function tests according to ranges for inclusion and exclusion defined in the protocol. Cognitive assessment was based on the Alzheimer's Disease Assessment Scale-cognitive subscale (participant), Neuropsychiatry Inventory (study companion), Bristol Activities of Daily Living Scale (study companion), Dementia Quality of Life (participant), and Dementia Quality of Life-Proxy (study companion). MMSE=Mini-Mental State Examination.

After randomisation, participants in both groups underwent an identical two-dose oral titration during the first 14 days of the intervention phase. In the first 7 days, patients received 25 mg of losartan orally or placebo, then 100 mg of the allocated treatment during days 8–14. After this intervention period, blood samples for safety assessment were taken and a 100 mg daily dose was maintained. The next assessment visit was at 3 months from randomisation and subsequent visits occurred every 3 months (ie, 6, 9, and 12 months) to facilitate provision of medication, pharmacovigilance, and compliance assessment by returned pill counts and repeat assessments when appropriate (figure 1). All main assessments (baseline, 6-month, and 12-month visits) were completed by both the participant and their study companion at in-person meetings, either at a home visit or at the clinical research centre, depending on participant preference. The 9-month visit was similar to the 3-month visit with the exception that no safety blood samples were obtained.

No dose modifications were allowed after randomisation and if any issues assessed by the local primary investigator to be related to the intervention occurred, patients were told to stop treatment, but were asked to remain in the study if possible. The intervention phase concluded after 12 months (ie, as close as possible to 52 weeks) after taking the first dose of the intervention or placebo, after which a minimum of 4 days washout commenced, to allow clearance of any residual intervention in participants for the final MRI. The final repeat assessments were done as close to the final MRI as possible, according to the assessment schedule. In summary, all cognitive, behavioural, and functional assessments were done at baseline, 6 months, and 12 months. All visits, including at 9 months, included blood pressure and intervention compliance assessments, as well as blood safety measures.

Cognitive assessments included the 11-item Alzheimer's Disease Assessment Scale-cognitive subscale (ADAS-Cog), the Neuropsychiatry Inventory, and the MMSE. Quality of life was self-reported by participants using the dementia quality of life (DEMQOL)measure and assessed by their study partner (DEMQOL-proxy), and activities of daily living were scored on the Bristol Activities of Daily Living Scale (BADLS) as summarised previously.^20^

MRI scans were acquired using either 1·5 T (n=44 at baseline; n=32 at follow-up) or 3 T (n=167 at baseline; n=139 at follow-up) MRI scanners with high-resolution (1 mm isotropic) 3-dimensional (3D) T1-weighted MPRAGE and all participants were scanned on the same magnet throughout the study period (ie, at baseline and end of the study). Semi-automated computerised methods derived brain structure volumes from single timepoint MRI and rates of atrophy from serial MRI, similar to other multicentre trials.^20^ Quality assurance of all scans and the quality assurance and editing of segmentations was done using MIDAS software (London, UK), whereas automated segmentations were done using Brain Multi-Atlas Propagation and Segmentation (BMAPS; London, UK) for whole brain areas and Similarity and Truth Estimation for Propagated Segmentations (STEPS; London, UK) for hippocampal regions, before manual checks and edits if needed.^20^ Longitudinal change following registration was measured using a Dementia Research Centre at University College London implementation of K-means normalised boundary shift integral (KN-BSI) for brains or double window KN-BSI for the hippocampus.^20^ BSI estimates atrophy by direct measurement of changes between aligned baseline and follow-up images, reducing reliance (compared with total brain volume measures) on segmentation accuracy and detecting sub-voxel shifts. Each participant underwent the RADAR scanning protocol (approximately 30 min). T2/FLAIR was also done to assess white matter hyperintensities, forming a prespecified substudy because of the limited availability across all centres, to explore whether the blood pressure lowering intervention affected white matter damage in participants and could predict 1-year cognitive decline.^16^

### Outcomes

The primary outcome was the measurement of change in brain volume, using volumetric MRI between baseline and after the 12-month visit. Volumetric MRI to measure brain atrophy is a recognised marker of Alzheimer's disease progression.^20^, ^24^

Secondary outcomes included (1) rates of probable Alzheimer's disease progression measured by changes from baseline in individual cognitive assessment scores, and quality of life assessed using DEMQOL; 2) change in the volume of white matter hyperintensities by MRI;^20^ 3) change in blood pressure; and 4) drug compliance and tolerability between study groups. Compliance was defined as participants having taken 80–120% of the intervention.^20^ Safety and adverse events were recorded through participant self-report; any observed deviations from blood safety measures according to protocol-defined ranges or other reported incidental events relevant identified in standard health care (eg, hospital admissions) were noted. Intended investigations in this study^20^ of the association between MRI measures of atrophy and the rate of cognitive decline, and in relation to cerebral blood flow, will be reported separately.

### Statistical analysis

Our sample size was calculated on the basis of findings from the use of volumetric MRI in the Alzheimer's Disease Neuroimaging Initiative (ADNI),^25^ which showed a mean rate of brain atrophy of 15**·**2 mL/year (SD 8**·**6). The study was powered to detect a relative difference between group atrophy rates of 25%, which we assert, on the basis of existing literature,^25^ is a clinically meaningful slowing of atrophy progression—equivalent to an absolute difference in annual total brain volume loss (ie, atrophy) of 3**·**8 mL. This rate was equivalent to a standardised effect size of 0·44 SD at 12 months, as previously described.^20^ We aimed to recruit and randomise 228 participants to obtain primary outcome data on at least 182 participants (assuming 20% loss to follow-up) for analysis, which would provide 84% power to detect our target difference of 3·8 mL/year in 12-month atrophy (therapeutic benefit) with a two-sided α level of 0**·**05.

Analysis and reporting of this trial followed a predefined statistical analysis plan, which was agreed with the Data Monitoring and Ethics Committee and confirmed with the Trial Steering Committee before the completion of data collection and analysis. Preliminary analyses summarised the trial population and compared descriptively the randomisation groups at baseline. Linear regression was used for the primary analysis to compare total brain volume and brain BSI reduction at 12-months’ follow-up between treatment groups, adjusted for baseline brain volume, minimisation variables (age group and Scheltens visual rating score groups), and recruitment site. The primary analysis was done by the intention-to-treat principle (ITT; ie, all participants were analysed in their randomised allocation groups, without imputation for missing data). The linear regression model results are presented as adjusted difference in means between the losartan and placebo groups alongside the associated 95% CIs and p values for comparison. Secondary outcomes were investigated using appropriate regression models adjusted for baseline value of the outcome being examined, minimisation variables, and recruitment site. Safety analysis was based on all participants but limited by the extent of adverse event self-reporting.

Several prespecified sensitivity analyses were done. First, assessment of the effect of missing data on the primary outcome was done using multiple imputation by chained equations method (MICE). This imputation model included all the variables in the primary ITT analysis, secondary outcomes (from each timepoint), and baseline variables associated with the missingness of the primary outcome. 20 imputed datasets were generated and combined using Rubin's rules, and the primary analysis model was then repeated using the imputed data. The second sensitivity analysis assessed the effect of treatment compliance on primary outcome using the allocation respecting method of complier average causal effects (CACE) via instrumental variable two-stage least-squares regression. Outcomes of those who complied with the intervention were compared with a group of compliers in the control group. We specified a priori the following potential exploratory analyses to assess effect modification on the primary outcome: baseline hypertension, baseline MMSE, baseline age, time since Alzheimer's disease diagnosis, baseline brain volume, and change in systolic blood pressure. A post-hoc analysis was also done to investigate for differences between aggregated and disaggregated MRI data (according to MRI scanner modality) for the primary outcomes.

We judged p values lower than 0·05 significant. All statistical analyses were done using STATA, version 15.

This trial is registered with the International Standard Randomised Controlled Trial Register, ISRCTN93682878, and the European Union Drug Regulating Authorities Clinical Trials Database, 2012–003641–15.

### Role of the funding source

The funder had no role in study design, data collection, data analysis, data interpretation, or writing of the report.

## Results

**Figure 2:**
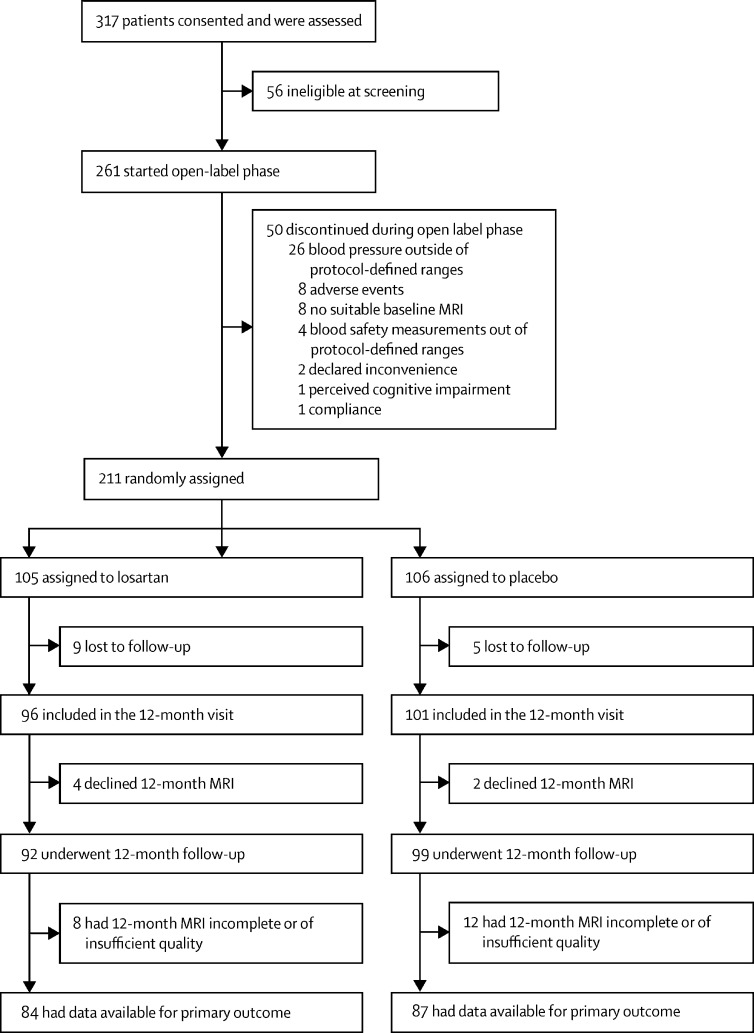
Trial profile

Between July 22, 2014, and May 17, 2018, 261 participants (and study companions) were recruited to the open-label phase of the study (figure 2). 211 patients were randomly assigned, 105 to the losartan group (intervention) and 106 to the placebo group (figure 1). Of 197 (93%) participants who completed the study, primary outcome data were available for 171 (81%) participants.

There were no significant differences in the patients’ baseline characteristics between treatment groups (table 1). About a third of participants in each study group were women, participants were predominantly white, and median age was similar between groups.

**Table 1:** Baseline characteristics

		**Placebo group (n=106)**	**Losartan group (n=105)**
Sex			
	Male	67 (63%)	60 (57%)
	Female	39 (37%)	45 (43%)
Ethnicity			
	White	106 (100%)	104 (99%)
	Other	0	1 (1%)
Age, years			
	<70	39 (37%)	39 (37%)
	70–79	42 (40%)	39 (37%)
	>79	25 (24%)	27 (26%)
Hypertensive		50 (47%)	47 (45%)
Education*, years		12 (11–16)	12 (10–16)
Systolic blood pressure†, mm Hg		136 (15)	138 (13)
Diastolic blood pressure†, mm Hg		78 (8)	79 (9)
Years since diagnosis		1·10 (0·69–2·43)	1·38 (0·64–2·29)
Total brain volume, mL		1036 (111)	1022 (99)
Total intracranial volume, mL		1459 (146)	1440 (140)
Lateral ventricle volume, mL		47 (35–64)	48 (35–69)
Total hippocampal volume, mL		5·0 (1·0)	5·2 (0·9)
Left hippocampal volume, mL		2·5 (0·5)	2·5 (0·5)
Right hippocampal volume, mL		2·6 (0·5)	2·6 (0·5)
Scheltens score			
	Absent or low (0–1)	62 (58%)	62 (59%)
	Moderate or severe (2–4)	44 (42%)	43 (41%)
ADAS-cog‡		19 (7)	20 (8)
MMSE§		22 (3)	22 (4)
NPI		6 (2–15)	8 (3–18)
BADLS		5 (2–9)	7 (2–13)
DEMQOL		96 (85–102)	96 (87–102)
DEMQOL-proxy		92 (83–99)	91 (82–100)

Data are n (%), median (IQR), or mean (SD). ADAS-cog=Alzheimer's Disease Assessment Scale–cognitive subscale. MMSE=Mini-Mental State Examination. NPI=Neuropsychiatric Inventory. BADLS=Bristol Activities of Daily Living Scale. DEMQOL=dementia quality of life. DEMQOL-proxy=carer-reported version of DEMQOL.

* In the intervention group, data were available for only 100 patients.

† Data were available for 84 patients in the placebo group and 83 patients in the losartan group.

‡ Data were available for 104 patients in the placebo group and 103 patients in the losartan group.

§ In the intervention group, data were available for only 103 patients.

The mean brain volume change from baseline to 12 months using the BSI (primary outcome) was 19·1 mL (SD 10·3) in the placebo group and 20·0 (SD 10·8) in the losartan group (table 2). The adjusted mean difference between groups was 1·23 mL (95% CI –1·72 to 4·19; p=0·41). The reductions in brain volume measured by BSI were very similar to those derived from subtracting total brain volume at 12 months from that at baseline and comparing between treatment groups (–2·29 mL, –6·46 to 0·89; p=0·14). Similar indications of deterioration were evident across both groups for all the cognitive assessments (ADAS-cog, Neuropsychiatry Inventory, and the MMSE), quality of life (DEMQOL, DEMQOL-proxy), and activities of daily living (BADLS), but there was no evidence of a difference between study groups (table 2). We found no evidence of differences in the volume of white matter hyperintensities in the substudy of 105 participants from 15 sites (placebo n=51, intervention n=54; p=0·70). Mean blood pressure levels were comparable at baseline between study groups, but after 12 months, the patients in the losartan group, unlike those in the placebo group, had greater reductions in systolic and diastolic blood pressure of 6·96 mm Hg (p<0·0001) and 3·59 mm Hg (p<0·0001), respectively. There was no strong correlation between the presence or absence of baseline hypertension and total brain volume (p=0·51) or total brain volume-BSI (p=0·57), nor of any interaction of these parameters with the volume of white matter hyperintensities (p=0·55). The compliance in the study was high (150 [88%] of 171) across all participants, but slightly lower in the losartan group (72 [86%] of 84) than the placebo group (78 [90%] of 87).

**Table 2:** Baseline, 12 months, and adjusted difference for primary and secondary outcomes by intervention group

	**Placebo group (n=106)**				**Losartan group (n=105)**				Adjusted mean difference*, mL (95% CI; n=171)	**p value**
	Baseline		12 months		Baseline		12 months			
	n	Mean (SD) or median (IQR)	n	Mean (SD) or median (IQR)	n	Mean (SD) or median (IQR)	n	Mean (SD) or median (IQR)		
**Primary outcome**										
Brain volume, mL	106	1036 (111)	87	1018 (113)	105	1022 (99)	84	1002 (98)	−2·29 (−6·46 to 0·89)	0·14
Brain BSI reduction, mL	106	..	87	19·1 (10·3)	105	..	84	20·0 (10·8)	1·23 (−1·72 to 4·19)	0·41
**Secondary outcomes**										
ADAS-cog	104	19 (7)	92	24 (12)	103	20 (8)	90	23 (12)	−0·52 (−2·71 to 1·66)	0·64
MMSE	106	22 (3)	97	19 (6)	105	22 (4)	95	19 (6)	−0·33 (−1·43 to 0·78)	0·56
NPI	106	6 (2–15)	99	8 (3–17)	105	8 (3–18)	92	8 (3–18)	0·88† (0·68–1·13)	0·30
BADLS	106	5 (2–9)	100	7 (3–14)	105	7 (2–13)	94	10 (3–17)	1·00† (0·83–1·21)	0·98
DEMQOL	106	96 (85–102)	95	94 (85–101)	105	96 (87–102)	91	96 (87–105)	0·98† (0·89–1·09)	0·74
DEMOL-proxy	106	92 (83–99)	98	93 (82–100)	105	91 (82–100)	92	93 (83–99)	1·43 (−1·43 to 4·28)	0·33
White matter hyperintensities	51	7354 (3418–20 019)	51	9793 (4788–20 263)	54	10 910 (2361–24 129)	54	11992 (2548–24 039)	0·99† (0·93–1·05)	0·70
Systolic blood pressure, mm Hg	84	136 (15)	98	139 (17)	83	138 (13)	95	133 (21)	−6·96 (−10·15 to −3·78)	<0·0001
Diastolic blood pressure, mm Hg	84	79 (8)	98	81 (9)	83	79 (9)	95	78 (13)	−3·59 (−5·29 to −1·89)	<0·0001

Data are n, median (SD), or median (IQR). BSI=boundary shift interval. ADAS-cog=Alzheimer's Disease Assessment Scale–cognitive subscale. MMSE=Mini-Mental State Examination. NPI=Neuropsychiatric Inventory. BADLS=Bristol Activities of Daily Living Scale. DEMQOL=dementia quality of life. DEMQOL-proxy=carer-reported version of DEMQOL.

* Adjusted for baseline measure of the outcome, minimisation variables, and centre.

† Ratio of geometric means.

Only one serious adverse event was reported in the open-label phase, which prompted withdrawal due to observed creatinine levels outside of protocol-specified ranges. In the intervention phase, there were comparable reported serious adverse events between the losartan (n=22) and the placebo groups (n=20). There were no apparent differences between the losartan and placebo groups concerning the numbers of adverse events (table 4 and appendix p 4). The most commonly reported serious adverse events were infections (six in losartan group *vs* one in the placebo group); mechanical injury (four *vs* four); neuropsychiatric (two *vs* three) and gastro-intestinal (one *vs* three). There was one death in each treatment group, both of which were deemed unrelated to the treatment (previously undiagnosed pancreatic cancer in the placebo group and anaemia and dehydration following admission to hospital for a femur fracture in the losartan group).

**Table 4:** Adverse events by follow-up timepoint and intervention group

		**Placebo group**					**Losartan group**				
		14 days (n=106)	3 months (n=106)	6 months (n=101)	9 months (n=100)	12 months (n=100)	14 days (n=104)	3 months (n=104)	6 months (n=101)	9 months (n=99)	12 months (n=96)
Any adverse event		32 (30%)	43 (41%)	46 (46%)	41 (41%)	45 (45%)	30 (29%)	51 (49%)	46 (46%)	45 (45%)	37 (39%)
Any serious adverse event		2 (2%)	4 (4%)	6 (6%)	4 (4%)	4 (4%)	2 (2%)	1 (1%)	9 (9%)	2 (2%)	6 (6%)
Serious adverse event											
	Mechanical injury	1 (1%)	0	0	2 (2%)	0	0	0	1 (1%)	2 (2%)	0
	Infection	0	0	0	0	1 (1%)	0	0	5 (5%)	0	1 (1%)
	Neuropsychiatric	0	1 (1%)	1 (1%)	1 (1%)	0	0	1 (1%)	1 (1%)	0	0
	Gastrointestinal	0	0	2 (2%)	0	1 (1%)	0	0	0	0	1 (1%)
	Other	1 (1%)	3 (3%)	3 (3%)	1 (1%)	2 (2%)	2 (2%)	0	2 (2%)	0	4 (4%)

Data are n (%). Additional information on adverse events is in the appendix pp 4, 5. Three serious adverse events are not included in this table, because they occurred before randomisation and, thus, the main phase of the study during which the primary outcomes are reported. Two of these were in participants during the open-label phase of the study but were included in the main trial (both randomised to intervention), and one participant who did not proceed to the randomised phase.

Analysis using MICE for missing data found no evidence of any intervention effect (adjusted difference in means –1·26, 95% CI –5·77 to 3·25, p=0·58). CACE analysis based on self-reported compliance differed little from the main results (adjusted difference in means –3·23, –7·14 to 0·69, p=0·11). We found no evidence of effect modification from baseline hypertensive status, baseline MMSE, baseline age, duration of Alzheimer's disease diagnosis, baseline brain volume, and change in systolic blood pressure (data not shown). For completeness, the primary outcome, according to MRI magnet strength (ie, 1·5 T *vs* 3·0 T) was checked to test for errors due to aggregating the data. Including a treatment group by scan modality interaction term provided no evidence of a difference in the effect of treatment on brain volume (interaction coefficient 0·43, 95% CI –9·45 to 10·31; p for interaction 0·93) or BSI between scan modality used (1·38, –6·57 to 9·34; p for interaction 0·73). The corresponding disaggregated outcome data are show in table 3).

**Table 3:** Main primary analysis according to treatment group and MRI scan modality

	**Baseline**			**12-month follow-up**		
	n	Brain volume, mL	BSI	n	Brain volume*, mL	BSI†
**1·5 T**						
Losartan group	22	1021 (100)	..	15	987 (84)	20·9 (10·8)
Placebo group	22	1051 (103)	..	17	1033 (109)	21·9 (11·6)
**3 T**						
Losartan group	83	1023 (100)	..	69	1005 (102)	19·8 (10·8)
Placebo group	84	1032 (114)	..	70	1015 (114)	18·4 (9·9)

Data are n or mean (SD). Repeat of main primary analysis including treatment group by scan modality (1·5 T *vs* 3 T) interaction term. BSI=boundary shift interval.

* p=0·93.

† p=0·73

## Discussion

We found that 12-months’ treatment with losartan in patients with clinically diagnosed mild-to-moderate probable Alzheimer's disease did not have any significant slowing in loss of brain volume measured by volumetric MRI, despite reduction in peripheral blood pressure, compared with placebo. Similarly, no therapeutic benefit was seen in hippocampal atrophy and ventricular volume, where annual change in hippocampi was between 2·5–5·0%, which is consistent with those previously reported over a similar duration.^26^ Sensitivity analyses that repeated the primary outcome analyses but accounted for missing data and compliance showed no substantial differences compared with the primary analyses. Similarly, there was no observable benefit on our secondary outcomes, including volume of white matter hyperintensities; we hypothesised that losartan could have an effect on this parameter given that this drug has been shown to improved cardiovascular outcomes,^17^ although our study was not adequately powered to show statistical differences for this secondary outcome.

Losartan was well tolerated even in normotensive patients, likely improved by the open-label phase whereby almost 80% of those who discontinued at this stage (from n=50) were normotensive. Yet, there was relative equivalency for participants with normotension in the placebo (47%) and losartan (45%) groups, and for serious adverse events or adverse events, highlighting the safety of the intervention. This notion is supported by comparable retention rates between the study groups.

The RADAR trial is the first randomised controlled trial, to our knowledge, to explore the potential therapeutic benefit of losartan compared with placebo on cognition and volumetric MRI measures of atrophy in participants with clinically diagnosed mild-to-moderate probable Alzheimer's disease who were either normotensive or hypertensive. Hajjar and colleagues^27^ recently reported a similar sized study (n=176) involving participants with hypertensive mild cognitive impairment who were treated over 12 months with candesartan, an ARA with a function similar to that of losartan. Participants on candesartan outperformed the ACE-I (lisinopril) for the primary outcome of executive function and secondary outcomes measured by Hopkins’ verbal learning-revised delay recall and retention measure, independent of blood pressure changes. However, as in RADAR, they found no evidence in their secondary outcome assessment of white matter hyperintensities between treatment groups by MRI. Another randomised controlled trial, NILVAD,^28^ that tested the efficacy of the calcium channel blocker nilvadipine in patients with hypertensive mild-to-moderate Alzheimer's disease, found no therapeutic effect. A small NILVAD single-centre substudy (n=44) despite not finding any evidence of change in either brain volume or the volume of white matter hyperintensities, reported increased hippocampal blood flow.

The RADAR trial recruited 93% of its intended target, had good overall compliance to an intervention that was as safe as placebo, and a higher than predicted (about 80%) retention rate. Reassuringly, the good mean to standard deviation ratio for BSI measurements showed that the 12-month treatment period was sufficient to detect differences in atrophy, similar to previous reports,^25^, ^26^ which would have detected our proposed clinically meaningful 25% relative difference between groups, had the intervention had an effect. Although longer follow-up would have been desirable, it might have been at the expense of higher drop-out rates or fewer primary outcome data because more participants might not complete the follow-up MRI scan.

Although there were some missing data, this amount was comparable in both groups. We found no evidence of benefits on white matter hyperintensities volume (table 2), irrespective of the previous existence of hypertension, although these analyses were exploratory and based on a subgroup of participants due to MRI scanner specifications at some sites. Our null findings might or might not reflect a type II error due to insufficient statistical power. Reassuringly, the intervention reduced blood pressure, proving that it was biologically active. A major benefit was the use of the open-label phase that reduced dropouts and unnecessary patient or study partner burden, sparing participants from undertaking additional baseline MRI scans and study visits if they were to discontinue early in the study and potentially preclude them from entry to other studies they might have been more suited to. This approach has since been recommended to provide reassurance, particularly for normotensive patients who might be anxious about taking an antihypertensive drug, as has been noted previously in dementia trials.^19^ Finally, many clinical studies now involve more in-depth biomarker analysis for the selection of participants, which was still in its infancy or prohibitively expensive when this study commenced, resulting in RADAR being a more naturalistic study design that might have introduced heterogeneity in the patient sample, although the evident robustness of our randomisation will hopefully have minimised any such effects.

It is possible that losartan did not penetrate the blood-brain barrier as much as anticipated; therefore, although peripherally active, as seen by the reductions in blood pressure in the losartan group, it was not able to act more directly in the brain where it was intended to reduce disease-related atrophy.

Our randomised controlled trial does not support evidence from observational studies and a recent systematic review^29^ that ARAs might offer preferential benefit over other forms of hypertension treatment regarding the incidence and progression of Alzheimer's disease. Other meta-analyses conclude that there are no such hypertension treatment differences over dementia incidence,^2^, ^9^, ^10^ although those findings and others agree that hypertension management is protective.^11^

The differences between the RADAR trial findings in patients with probable Alzheimer's disease and those of Hajjar and colleagues^27^ in patients with hypertensive mild cognitive impairment highlight how study size, inclusion criteria and patient group, follow-up period, and choice of outcome measures^1^ will continue to be vital in determining whether ARAs have potential as treatments in the development or progression of cognitive decline and dementia. The opposing findings to date highlight the importance of other studies yet to complete, which are also testing the angiotensin hypothesis of Alzheimer's disease (ie, that excess angiotensin II signalling resulting from imbalance in the brain RAS contributes to the pathogenesis of Alzheimer's disease)^6^ by investigating various ARAs in populations with no cognitive impairment but at risk of developing dementia (ie, HEART [NCT02471833]^30^ and the rrAD study [NCT02913664]) or with mild cognitive impairment (CEDAR [NCT02646982]).

## Data sharing

All data requests should be submitted to the corresponding author for consideration. Access to available anonymised data might be granted following review.

## Declaration of interests

PGK reports grants from the National Institute of Health Research (NIHR), during the conduct of the study; being a non-funded co-investigator of the ongoing Alzheimer's Association-funded HEART phase 1b study of telmisartan and its use as an intervention against the renin–angiotensin system in African American people at risk of developing dementia by parental history; and having previously undertaken advisory work for Novartis in their development and intended trialling of dual acting inhibitors of Angiotensin Receptor Blockers and neprilysin (LCZ696) for the treatment of heart failure. NT, BH, LJ, SLB, IBM, CN, NJT, PSB, JAL, PP, JT, DLT, IW, and YBS report grants from the NIHR, during the conduct of the study. JB reports grants from Alzheimer's Research UK, outside the submitted work. CHS reports grants for the Alzheimer's Society, during the conduct of the study. EC reports grants from the NIHR, during the conduct of the study; and received payment from Biogen, Novartis, and Union Chimique Belge for providing educational resources or consultancy around Alzheimer's Disease trials. NCF reports other funding from Roche, personal fees from Biogen, and non-financial support from Lilly and Ionis, outside the submitted work. AW and H-JM declare no competing interests.
