# Supplementary material for: Safety and efficacy of losartan for the reduction of brain atrophy in clinically diagnosed Alzheimer's disease (the RADAR trial): a double-blind, randomised, placebo-controlled, phase 2 trial
Source: Lancet Neurol. 2021 Nov;20(11):895–906. doi: 10.1016/S1474-4422(21)00263-5 (PMC8528717; doi:10.1016/S1474-4422(21)00263-5)
Supplement: Supplementary appendix [file mmc1.pdf]

THE LANCET
Neurology

Supplementary appendix

This appendix formed part of the original submission and has been peer reviewed. We post it as supplied by the authors.

Supplement to: Kehoe PG, Turner N, Howden B, et al. Safety and efficacy of losartan for the reduction of brain atrophy in clinically diagnosed Alzheimer's disease (the RADAR trial): a double-blind, randomised, placebo-controlled, phase 2 trial. *Lancet Neurol* 2021; **20:** 895–906.

Supplemental Tables
to
Safety and efficacy of losartan for the reduction of brain atrophy in clinically diagnosed Alzheimer's disease (the RADAR trial): a phase 2 double blind, randomised, placebo-controlled trial

**Contents**	**Page Number**
Supplemental Table 1 – Summary of Amendments to Protocol	**1**
Supplemental Table 2 - Comparisons of primary outcome according to Hippocampal and Lateral Ventricle volumes	**3**
Supplemental Table 3 - Serious Adverse Events (SAE) according to Treatment Arm	**4**

Supplemental Table 1 – Summary of Amendments to Protocol

Trial Amendment Type & Number	Protocol Version No.	Date Issued	Author(s) of changes	Summary of changes made
Substantial Amendment 1 (S/A1)	3.0	03/06/2013	Patrick Kehoe	Revisions made for clarity and to ensure consistency with the sponsor's SOPs and previous recommendations made by the ethics committee See S/A1 summary dated 03/06/2013
Substantial Amendment 2 (S/A2)	4.0	18/07/2013	Patrick Kehoe	Addition of information pertaining to pilot imaging procedures
Minor Amendment 2	4.1	23/07/2013	Patrick Kehoe	Minor clarifications added after review by TSC, DMEC and TMG
Substantial Amendment 3 (S/A3)	5.0	28/11/2013	Patrick Kehoe	Change to minimisation details and eligibility criteria. Clarification that an additional phonecall is required to provide the outcome of the screening blood test.
Minor Amendment 3	5.1	09/04/2014	Patrick Kehoe	Minor corrections to ensure consistency with other documentation/text e.g. timing of blood tests
Substantial amendment 6 (S/A 6) NB substantial amendments 4&5 were approved by the MHRA only due to	6.0	07/10/2014	Patrick Kehoe	Removal of exclusion criteria for severe hippocampal atrophy and inclusion of option to recruit via primary care.

change in brand of the IMP and shelf-life extension.				
Minor Amendment 4	6.0	07/10/2014	Patrick Kehoe	Submission for review of revised poster to be used in recruitment from primary care.
Minor Amendment 5	6.1	14/11/2014	Patrick Kehoe	Removal of residual references in protocol text and Appendix I of protocol to severe hippocampal atrophy as exclusion criteria that were missed in Substantial amendment 6 (S/A 6)
Minor Amendment 6	6.1	26.02.2015	Patrick Kehoe	Formatting changes to the open label diaries for BP and medication and change to the reply slip to include GP surgery.
Minor Amendment 7	6.1	12.05.2015	Patrick Kehoe	Inclusion of RADAR on 'Join Dementia Research' (JDR)
Substantial Amendment 7 (S/A7)	7.0	03/12/2015	Patrick Kehoe	Change of eligibility criteria for MMSE range from 18-28 to 15-28.
Substantial Amendment 8 (S/A8)	8.0	04.03.2016	Patrick Kehoe	Inclusion of an embedded qualitative component.
Substantial Amendment 9 (S/A9)	9.0	19.07.2017	Patrick Kehoe	Clarification of consent process. Inclusion of SPECT as a considered scan option for screening, and similarly use of ACE-R cognitive assessment scores as a reference point for screening of potentially eligible participants. Clarification of baseline Blood pressure exclusion criteria. Clarification of assessment timings. Replace ‘informant’ with ‘companion’
Substantial Amendment 10 (S/A10)	10.0	11.10.2017	Patrick Kehoe	Correction to blood pressure exclusion criteria section 7.2.

Supplemental Table 2 – Comparisons of primary outcome according to Hippocampal and Lateral Ventricle volumes

	Placebo				Intervention				Regression analysis	
	Baseline		12 months		Baseline		12 months			
	n	Mean (SD)	n	Mean (SD)	n	Mean (SD)	n	Mean (SD)	Adjusted* mean diff (95%CI)	p-value
**LHV**	106	2.5 (0.5)	87	2.4 (0.5)	105	2.5 (0.5)	84	2.5 (0.5)	0.01 (-0.01, 0.03)	0.337
**LHV BSI**	-	-	87	0.11 (0.06)	-	-	84	0.11 (0.08)	-0.01 (-0.03, 0.01)	0.385
**RHV**	106	2.6 (0.5)	87	2.5 (0.5)	105	2.6 (0.5)	84	2.6 (0.5)	0.01 (-0.003, 0.03)	0.112
**RHV BSI**	-	-	87	0.12 (0.06)	-	-	84	0.11 (0.08)	-0.01 (-0.03, 0.01)	0.231
**THV**	106	5.0 (1.0)	87	4.9 (0.9)	105	5.2 (0.9)	84	5.0 (0.9)	0.02 (-0.01, 0.05)	0.144
**LVV**	106	47 [35, 64]	86	50 [40, 67]	105	48 [35, 69]	83	51 [40, 78]	0.99^{+} (0.98, 1.01)	0.443
**LVV BSI**	-	-	86	5 [3, 7]	-	-	83	5 [3, 8]	-0.45 (-1.45, 0.56)	0.380

Legend. LHV= left hippocampus volume; RHV= right hippocampus volume, THV= total hippocampal volume; BSI = Boundary Shift Integral; LVV = lateral ventricle volume,. *Adjusted for minimization variables (age and Scheltens score categories), baseline value of the outcome and centre. $^{+}$Ratio of geometric means.

Supplemental Table 3 - -Serious Adverse Events (SAE) according to Treatment Arm

SAE Number	Brief Summary	Arm
**Cancer**		
SAE002	Participant died pancreatic cancer	Placebo
**Cardio-circulatory**		
SAE003	Prolonged syncopal episode	Placebo
SAE018	Fainting and bradycardia	Placebo
**Dermatological**		
SAE030	Rash around armpits and right side of the abdomen	Intervention
**Endocrine and metabolic**		
SAE013	Development of Diabetes	Intervention
**Gastrointestinal**		
SAE016	Constipation and prolapse	Placebo
SAE033	Diverticulitis	Placebo
SAE027	Stomach pain and breathing difficulty	Placebo
SAE024	Urinary retention and diarrhoea and vomiting	Intervention
**Haematological/thrombosis**		
SAE040	Admission 3 days Right sided chest pain. Discharged with anticoagulant meds.	Placebo
**Hepatic**		
SAE017	Collapse Raised ALT 100 (0-55)	Intervention
SAE023	Grossly raised AST serum level	Placebo
SAE043	Safety bloods results outside of the safety range	Intervention
**Infection**		
SAE007	Hospital admission. Sepsis	Intervention
SAE019	Cellulitis left leg	Intervention
SAE020	Cellulitis left leg flare up	Intervention
SAE029	Recurrent bronchitis	Intervention
SAE039	Dizzy spell, tremor and cold hands. Discharged with antibiotics.	Intervention
SAE041	Chest infection	Placebo
SAE042	Lower respiratory tract infection	Intervention
**Mechanical injury**		
SAE006	Fall	Placebo
SAE008	Fainting	Placebo
SAE010	Participant died. Admitted with femur fracture, dehydration and anaemia. Unrelated to trial	Intervention
SAE011	Fall	Intervention
SAE014	Fainting	Intervention
SAE021	Falls	Intervention
SAE035	Fall	Placebo

Continued on next page

Continued from previous page

SAE Number	Brief Summary	Arm
**Neuropsychiatric**		
SAE012	Found slumped on sofa unable to sit up, visual hallucinations	Placebo
SAE004	Seizures	Intervention
SAE026	Seizure	Placebo
SAE032	Vivid delusions and aggressive outburst	Placebo
SAE034	Temp admission to Dementia Assessment Unit	Intervention
**Other**		
SAE031	Ankle swelling (oedema)	Placebo
SAE001	Brain pathology	Intervention
SAE005	Persistent nausea, dizziness and headache	Placebo
SAE009	Mobility problems and unable to get out of bath	Intervention
SAE015	Overdose of dementia medication	Intervention
SAE025	Food poisoning	Intervention
SAE028	Collapse	Intervention
SAE036	Elective surgery (throat biopsy)	Placebo
SAE037	Hospital admission knee pain. Arthritis	Placebo
SAE038	Collapse without loss of consciousness	Placebo
**Renal**		
SAE022	Increased creatinine	Open Label
